# Dietary Intake Is Associated with Phthalate Body Burden in a Nationally Representative Sample

**DOI:** 10.1289/ehp.0901712

**Published:** 2010-04-14

**Authors:** Justin A. Colacino, T. Robert Harris, Arnold Schecter

**Affiliations:** 1 Department of Environmental Health Sciences, University of Michigan School of Public Health, Ann Arbor, Michigan, USA; 2 University of Texas School of Public Health, Dallas Regional Campus, Dallas, Texas, USA

**Keywords:** diet, metabolites, NHANES, phthalates, United States

## Abstract

**Background:**

Phthalates are compounds that are used in a wide range of consumer products. However, the contribution of dietary intake to phthalate exposure has not been well defined.

**Objective:**

The objective of this study was to assess the contribution of different food types to phthalate exposure. Phthalates are chemicals of concern because of the high levels measured in people and the environment, as well as the demonstrated toxicity in animal studies and limited epidemiological studies. Previous research, although limited, has suggested that phthalates contaminate food in various countries.

**Methods:**

We conducted an exploratory analysis of data collected as part of the 2003–2004 National Health and Nutrition Examination Survey (NHANES). Associations between dietary intake (assessed by a 24-hr dietary recall) for a range of food types (meat, poultry, fish, fruit, vegetable, and dairy) and phthalate metabolites measured in urine were analyzed using multiple linear regression modeling.

**Results:**

We found that metabolites of di-(2-ethylhexyl) phthalate (DEHP) and high-molecular-weight phthalate metabolites were associated with the consumption of poultry. Monoethyl phthalate, the metabolite of diethyl phthalate (DEP), was associated with vegetable consumption, specifically tomato and potato consumption.

**Discussion:**

These results, combined with results from previous studies, suggest that diet is an important route of intake for phthalates. Further research is needed to determine the sources of food contamination with these toxic chemicals and to describe the levels of contamination of U.S. food in a large, representative U.S. sample.

Phthalates are a family of chemicals that have been approved for use in a wide range of consumer products, including building materials, personal cosmetics, pharmaceuticals, nutritional supplements, solvents, adhesives, paints, lacquers, insecticides, cleaning materials, children’s toys, and food packaging (Schettler 2006). The annual global use of phthalates is estimated to exceed 3 million metric tons per year (Schettler 2006). Phthalates have been used for > 50 years to increase the plasticity of rigid materials such as polyvinyl chloride (PVC).

Toxicological studies of phthalates and related compounds have established potential health effects of exposure, particularly endocrine disruption. In animal studies, researchers have found an association between phthalate exposure and the induction of testicular toxicity in rats and guinea pigs as well as male reproductive tract lesions such as seminiferous epithelial degeneration (Barlow et al. 2004; Gray et al. 1982). Phthalate exposure also has effects on the female reproductive system, including decreased estradiol production, prolonged estrous cycles, and no ovulation in adult rats (Davis et al. 1994; Lovekamp-Swan and Davis 2003). In a three-generation phthalate exposure study, Pereira et al. (2007) found significant increases in liver enzymes [alanine aminotransferase (ALT) and aspartate aminotransferase (AST)] over the three generations and decreases in serum cholesterol, liver glutathione, and glutathione reductase, especially in the F2 generation. Effects on sexual health and development have been observed in recent human studies. Phthalate exposure was associated with decreased anogenital distance in male infants, which was found to be significantly correlated with penile volume and cryptorchidism (Swan et al. 2005). A study using the National Health and Nutrition Examination Survey (NHANES) data from 1999–2002 found an association between urinary phthalate metabolites and increased waist circumference and insulin resistance (Stahlhut et al. 2007). Additionally, increased phthalate body burden, particularly of one metabolite, mono-2-ethylhexyl phthalate (MEHP), was associated with decreases in testosterone and estradiol in adult men (Meeker et al. 2009). Levels of phthalates in house dust have been associated with rhinitis, eczema, and asthma in humans (Bornehag et al. 2004).

Typically, phthalate body burden is estimated through measurement of phthalate metabolites in the urine. The half-life of the parent phthalates is very short, and high levels of contamination in laboratories present technical difficulties when analyzing for phthalates (Lorber et al. 2010). Silva et al. (2004), assessed body burdens of phthalate metabolites for a portion of the U.S. population in NHANES. In this study, they found that children had significantly higher levels of certain phthalate metabolites, monobutyl phthalate, monobenzyl phthalate (MBzP), and MEHP than did adults; however, they had significantly lower levels of monoethyl phthalate (MEP), likely because of the use of MEP in consumer products such as cosmetics, which are used more by adults than by children. Another study compared the levels of phthalate metabolites in infants and their mothers and found that, on average, creatinine-adjusted levels of phthalate metabolites were higher in infants than in their mothers (Sathyanarayana et al. 2008). Creatinine adjustment has been used to normalize excretion of phthalate metabolites and many other contaminants (Jackson 1966). Given a daily rate of elimination of creatinine, one can estimate a full day’s elimination of the phthalate metabolite. With other considerations, this daily elimination can be used to estimate the intake that may have been associated with this measurement. A study of a Danish–Finnish cohort of mothers found phthalate metabolites in all samples of breast milk that were tested (Main et al. 2006).

Phthalates have been used widely as plasticizers in food packaging, and as such, diet is considered a major route of exposure (Schettler 2006). Research on the levels of phthalates in food is limited because the accurate determination of phthalate levels by a laboratory is severely complicated by the ubiquitous nature of phthalate contamination (Fromme et al. 2007). Phthalates have been measured previously in NHANES, beginning in NHANES III, where levels of phthalate metabolites were measured in samples collected from 289 adults in 1988–1994 (Blount et al. 2000), to more recent studies that have greatly expanded the sample population [Centers for Disease Control and Prevention (CDC) 2005, 2009; Silva et al. 2004].

Although research suggests that dietary intake is a significant route of exposure for phthalates, because of elevated levels detected in food samples (Fromme et al. 2007; Tsumura et al. 2001, 2003), few research studies have been conducted to quantify how dietary intake correlates with body burden of these toxic chemicals.

The primary aim of this study was to analyze the relationship between meat, dairy, fish, fruit, and vegetable consumption and total phthalate body burden in the United States using data from NHANES 2003–2004 (CDC 2010), an ongoing health and nutrition survey of the U.S. population. The secondary aim of this study was to analyze the relationship between meat, dairy, fish, fruit and vegetable consumption and body burden of individual phthalate metabolites, as well as the sums of metabolites of high‐ and low-molecular-weight phthalates.

## Materials and Methods

### Data set

The NHANES is an ongoing survey of the civilian, noninstitutionalized U.S. population designed to monitor the health status of adults and children [Centers for Disease Control and Prevention (CDC) 2010]. The survey combines questionnaire data with physical examinations as well as biomonitoring of chemical contaminants in blood and urine. NHANES is administered by the National Center for Health Statistics, and the biomonitoring is performed by the National Center for Environmental Health, both divisions of the CDC.

NHANES typically samples around 5,000 individuals each year using a complex, multistage probability sampling design with oversampling of populations that might be at greater health risk. Although data are collected continuously, data sets are reported in 2-year cycles to ensure that the sample size is sufficiently large. In the NHANES 2003–2004 survey, phthalate metabolites were measured in urine from 2,697 individuals ≥ 6 years old. Data for both measurements of phthalate metabolites and dietary intake were available for 2,374 individuals. There was no significant difference in age or sex between the individuals who had dietary intake data and those who did not. The proportion of white individuals was higher in the group that had dietary data than in the group that did not.

### Dietary intake

Dietary intake was assessed in NHANES using two 24-hr dietary recall face-to-face interviews, a 24-hr dietary recall conducted by telephone 3–10 days after the interview, and a food frequency questionnaire. Instructions for the interviews were provided orally during the first interview session in English, Spanish, or both languages. During both reporting sessions, information related to types of foods and beverages consumed, portions, and meal times was collected. The 24-hr dietary recall questionnaire gathered information about food type and portions consumed, and the food frequency questionnaire asked about frequency of food consumption but not about portion size. The food frequency questionnaire was distributed to enrolled participants who completed at least one 24-hr dietary recall interview. Because of the short half-life of phthalates in the human body, which are estimated to be on the order of hours, dietary intake was estimated using only the first 24-hr dietary recall, which assessed dietary intake for the day before urine was collected for phthalate measurements.

The individual foods measured in the dietary intake interview were grouped based on the MyPyramid equivalents [U.S. Department of Agriculture-Agricultural Research Service (USDA-ARS) 2010]. The groups of interest for this analysis were milk, meat and beans, vegetables, and fruit. Subgroups of interest were total dairy foods (milk, yogurt, and cheese); meat (beef, pork, veal, lamb, and game); frankfurters, sausage, and luncheon meats (made from meat or poultry); poultry (chicken, turkey, and other); fish and shellfish high in n-3 fatty acids; fish and shellfish low in n-3 fatty acids; eggs; vegetables; legumes; and fruit.

### Chemical analyses

Chemical analyses of phthalates were conducted as reported previously (Kato et al. 2005; Sjodin et al. 2004). Phthalate metabolites are reported individually in the NHANES data set. Any phthalate metabolites that were detected in fewer than 60% of the samples were excluded from future analyses, because of the inability to calculate geometric means for these congeners, as has been reported previously in NHANES analyses (Sjodin et al. 2008). Total phthalate metabolite levels were calculated for each individual by summing the levels of the analytes that met this condition. Levels that were below the limit of detection were estimated by dividing the limit of detection for that specific analyte by the square root of two. Additionally, high– and low-molecular-weight phthalate metabolite totals were calculated, because low– and high-molecular-weight phthalates are used in different classes of commercial products and thus may represent different routes of exposure. Low-molecular-weight phthalates are defined as those with molecular weight < 250 g/mol (Wolff et al. 2008). Additionally, associations between the sum of the four di-(2-ethylhexyl) phthalate (DEHP) metabolites [mono-2-ethyl-5-carboxypentyl phthalate (MECPP); mono-(2-ethyl-5-hydroxyhexyl) (MEHHP); MEHP; and mono-(2-ethyl-5-oxohexyl) phthalate (MEOHP)] and dietary intake were also calculated. Phthalate metabolites were analyzed on a creatinine-adjusted basis.

### Demographic variables

The demographic variables we analyzed included age, sex, ethnicity, and body mass index (BMI; the weight of an individual in kilograms divided by the square of their height in meters). A BMI of < 18.5 is considered underweight, 18.5–25 is considered normal weight, 25–30 is overweight, and > 30 is obese. In the multiple regression analyses used in this study, we treated BMI as a continuous variable.

### Data analysis

Food consumption, using the MyPyramid equivalents (USDA-ARS 2010), was correlated with total phthalate metabolite concentrations, total low and high-molecular-weight phthalate metabolite concentrations, and levels of individual phthalate metabolites. Three stages of data analyses were conducted. First, preliminary (or univariate) analysis was conducted to find mean, median, and ranges of the sample’s demographics, dietary characteristics, and body burden of phthalate metabolites based on sample weights. Means, geometric means, and medians of individual and total phthalate metabolites were also calculated based on weighted data. Phthalate metabolite levels were found to be highly skewed, thus log-transformed values of these variables were used in all subsequent bivariate and multivariable analyses.

Second, we assessed relationships between dietary intake of individual food types and measured levels of individual phthalate metabolites with bivariate analyses, including correlations and bivariate linear regression models. The dependent variable in these analyses was a measured chemical level, that is, individual phthalate metabolites, low– or high-molecular-weight metabolites, DEHP metabolites, or total metabolites. The total servings of one MyPyramid food subgroup (USDA-ARS 2010) were included in each model. All regression models were built using weighted data. Finally, for each association that was statistically significant in the bivariate analyses (*p* < 0.05), we constructed a multiple linear regression model, including that food type and the covariates age, BMI, sex, and ethnicity. Age and BMI were modeled as continuous variables, and sex and ethnicity were modeled categorically (Table 1).

We performed the data analysis with the survey procedures using SAS software (version 9.2; SAS Institute, Cary, NC), with the appropriate sample characteristics (strata, clusters, and weights), as described in the NHANES documentation (CDC 2010).

## Results

### Univariate analysis

Most individuals (52.4%) in the NHANES sample with food and phthalate data were female. The demographic distribution of the NHANES participants is displayed in Table 1. The age of NHANES participants ranged from 6 to 85 years old (weighted mean = 39.0 years). The BMI ranged from 13.0 to 58.6 (weighted mean = 26.8 kg/m^2^). The percentage of individuals who were classified as underweight, normal weight, overweight, and obese is shown in Table 2.

Table 3 presents the dietary intake of the food groups we analyzed. There was a wide range of meat consumption, from 0 ounce equivalents to 25.5 ounce equivalents consumed. The consumption of fish and poultry was also quite variable, with ranges of 0–14.3 and 0–17.7 ounce equivalents consumed, respectively. The median cups of fruit consumed was 0.6, whereas the median consumption of vegetables (excluding legumes) was 1.1.

Ten phthalate metabolites were detected in the urine of > 60% of the population: MECPP; mono*-n-*butyl phthalate (MnBP); mono-(3-carboxypropyl) phthalate (MCPP); MEP; MEHHP; MEHP; mono*-n-*methyl phthalate (MnMP); MeOHP; and MBzP. The measured levels of unadjusted phthalate metabolites are presented in Table 4, and the levels of creatinine-adjusted phthalate metabolites are shown in Table 5. MEP, a metabolite of diethyl phthalate (DEP), was detected at the highest concentration in the population (geometric mean 194.4 ng/mL; range, 3.5–46,347). Mono-(2-ethyl-5-carboxypentyl) phthalate (MECCP), a metabolite of DEHP, was detected at the next highest concentration (geometric mean 35.3 ng/mL; range, 0.2–2,887). The sum of the low- molecular-weight phthalate metabolites, MEP and MnMP, had a geometric mean of 201.8 ng/mL (range, 4.2–46,359). The sum of the high-molecular-weight phthalate metabolites, MECPP, MnBP, MCPP, MEHHP, MEHP, mono-isobutyl phthalate (MiBP), MEOHP, and MBzP, had a geometric mean of 137.4 ng/mL (range, 2.1–6,616). Total phthalates (the sum of both low– and high-molecular-weight phthalate metabolites) had a geometric mean of 437.9 ng/mL (range, 8.9–47,586).

Adjusting for creatinine concentration did not affect which phthalate metabolites were detected at the highest levels. MEP still had the highest geometric mean concentration at 184.3 μg/g creatinine (range, 1.9–29,986). The low-molecular-weight phthalates had a geometric mean creatinine-adjusted concentration of 190.5 μg/g creatinine (range, 2.1–29,987), whereas the high-molecular-weight phthalates had a geometric mean creatinine-adjusted concentration of 129.7 μg/g creatinine (range, 0.7–4,901). Total phthalates had a geometric mean creatinine-adjusted concentration of 413.3 μg/g creatinine (range, 2.8–30,119).

### Bivariate analysis

Scatterplots with LOESS curves did not show outliers or curvilinear relationships. Bivariate linear regression models estimated the relationship between consumption of single food groups and phthalate levels. Food types that had a statistically significant (*p* < 0.05) association were included in multiple regression analyses that adjusted for demographic covariates. The statistically significant results of the bivariate and multiple regression analyses can be seen in Table 6. Nonsignificant results of bivariate and multiple regression analyses can be seen in Supplemental Material, Table 1 (doi:10.1289/ehp.0901233). The regression coefficients shown describe the change in log-transformed creatinine-adjusted phthalate metabolite level associated with consumption of one additional serving of food.

In bivariate analyses, total dairy consumption was significantly associated with MCPP. Poultry consumption was a significant predictor of levels of all of the individual DEHP metabolites, MEHP, MEHHP, MEOHP, MECCP, total DEHP metabolites, as well as high-molecular-weight phthalate metabolites and total phthalate metabolites. Egg consumption was also significantly associated with urinary MEHP levels. High-molecular-weight phthalates, particularly DEHP, are lipid soluble (Dickerson 1997). Despite the high fat content in meat, consumption was associated with MEP levels, the metabolite of DEP, as well as with low-molecular-weight phthalate metabolites, likely because of the association with MEP. Vegetable consumption was also significantly associated with MEP levels, which was one of the strongest effects measured. To further examine the relationship between vegetable intake and MEP levels, we calculated the association between tomato and potato consumption and MEP levels. Consumption of both of these vegetables was associated with MEP levels. Additionally, tomato consumption was associated with low-molecular-weight phthalate metabolites, likely because of the strong association between tomato consumption and MEP levels. Fruit consumption was associated with levels of MnMP, a metabolite of dimethyl phthalate, a low-molecular-weight phthalate. Additionally, fruit consumption was found to be inversely associated with high-molecular-weight phthalate metabolites and DEHP metabolites. Fish consumption was associated with levels of MiBP, a metabolite of diisobutyl phthalate, a high-molecular-weight phthalate. Levels of MnBP were not found to be significantly associated with any of the dietary variables.

### Multivariable analysis

To adjust for possible covariates that may have confounded the effects observed in the bivariate regression analyses, we also performed multiple regression analyses. In these analyses, other variables included in the model were age, ethnicity, sex, and BMI. Phthalate levels were previously shown to be associated with BMI (Hatch et al. 2008). The regression coefficient for the association between the log-adjusted creatinine-adjusted DEHP metabolites and poultry consumption in the multivariable model was 0.055, suggesting that an increase of one ounce of poultry per day is associated with an increase in DEHP metabolite levels of approximately 5.7%.

## Discussion

This analysis demonstrates that there is an association between the reported consumption of different food types and the levels of phthalate metabolites in urine of study participants. Previous research has established that many commonly consumed food types are contaminated with persistent organic pollutants, although the literature is sparse for phthalate levels in food, because of the difficulties of analyzing parent phthalate compounds.

A study of homogenized duplicated diet samples collected over the course of 7 days from 50 total study subjects (27 women, 23 men) age 14–60 years old from Germany found that dietary intake of DEHP was significantly correlated with levels of DEHP metabolites measured in urine (Fromme et al. 2007). The median daily intake of DEHP was estimated to be 2.4 μg/kg body weight (95th percentile estimate: 4.0 μg/kg body weight), whereas the median daily intake of di*-n-*butyl phthalate (DnBP) was estimated to be 0.3 μg/kg body weight (95th percentile estimate: 1.4 μg/kg body weight). The median daily intake of di-isobutyl phthalate (DiBP) was estimated to be 0.6 μg/kg body weight (95th percentile estimate, 2.1μg/kg body weight). However, although DEHP intake was significantly correlated with levels of DEHP metabolites measured in study participants, similar correlations were not observed for DnBP and DiBP metabolites. In the current study, creatinine-adjusted levels of MEHP (a metabolite of DEHP) were significantly associated with poultry consumption; total meat, poultry, and fish consumption; and discretionary solid fat consumption in multivariate regression models. Creatinine-adjusted levels of another DEHP metabolite, MECPP, were also significantly associated with discretionary solid fat consumption in multivariate regression models. Creatinine-adjusted levels of MEHHP, also a DEHP metabolite, were significantly associated with poultry and discretionary solid fat consumption. Similar results were also observed for creatinine-adjusted MEOHP, a DEHP metabolite as well. The results from the Fromme et al. study (2007), as well as this study, point to diet as a significant route of exposure to DEHP, mostly likely from meat consumption or consumption of solid fats.

A research group from Japan examined the levels of phthalate esters in duplicate diet samples on two separate occasions (Tsumura et al. 2001, 2003). In the first study conducted in 1999, 1-week duplicate diet samples were collected for 63 patients in three hospitals from three geographically distinct prefectures in Japan (Tsumura et al. 2001). DEHP was also detected at the highest levels in these samples, with concentrations ranging from 10–4,400 ng/g wet weight (ww). There was a significant difference between mean levels of DEHP detected in the three hospitals, with two of the hospitals having considerably higher levels (384 ng/g and 478 ng/g) compared with 46 ng/g. The authors hypothesized that the elevated levels in two of the hospitals were due to the use of disposable PVC gloves, which used DEHP in manufacturing, during food preparation. In a follow-up study, Tsumura et al. (2003) examined levels of phthalates in duplicate diet samples that were collected in 2001after the use of DEHP in PVC gloves was banned. Once again, the researchers collected week-long duplicate diet samples from 63 patients at the same 3 hospitals. DEHP levels in these samples were detected at significantly lower levels than in the previous study, with a range of 6–675 ng/g ww compared with 10–4,400 ng/g ww in the previous study. The lower levels of phthalates detected in this study support the hypothesis of Tsumura et al. (2003) that the PVC gloves used in food preparation were a substantial source of phthalates in the total diet samples. The estimated daily average intake of DEHP was 160 μg/day. Nondetected levels in this study were estimated using half of the detection limit, which ranged from 0.1–15.6 ng/g ww, which could overestimate the daily phthalate intake because of the high detection limit. Tsumura et al. (2003) also measured levels of other phthalates whose metabolites were measured in the present study: di-*n*-butyl phthalate (DBP), benzylbutyl phthalate (BBP), dicyclohexyl phthalate (DCHP), and diisononyl phthalate (DINP). DBP was detected above the limit of quantification (LOQ) in 12 of 63 samples tested, with detected values ranging from 4.5–27.1 ng/g ww. BBP was detected above the LOQ in 29 of 63 samples, with detected values ranging from 0.9 to 27.1 ng/g ww. DCHP was not quantified in any samples. DINP was detected above the LOQ in 2 of 63 samples; one sample was 10 ng/g ww, the other 24 ng/g ww. The results of both studies suggested that diet is likely a significant route of exposure to phthalates (Tsumura et al. 2001, 2003).

A study conducted in Denmark examined the levels of DEHP, DBP, and BBP in total diet samples, infant formula, and baby food (Petersen and Breindahl 2000). DEHP was detected in 11 of 29 total diet samples above the LOQ, with an estimated average concentration in all samples ranging from 110 to 180 ng/g ww. DBP was detected above the LOQ in 6 of 29 total diet samples, with an estimated average concentration in all samples ranging from 90–190 ng/g ww. BBP was detected above the LOQ in 8 of 29 diet samples, with an estimated average concentration in all samples ranging from 17–19 ng/g ww. Plasticizers were detected in 6 of 11 baby food samples tested as well as 8 of 11 infant formula samples. DEHP was measured at the highest levels in both individual baby food and infant formula samples, 0.63 μg/g ww and 0.06 μg/g ww, respectively. Overall, DEHP was estimated to be consumed at the highest levels through diet, with an estimated intake of 0.30 mg/day, whereas the estimated daily consumption of DBP was 0.29 mg/day and 0.03 mg/day for BBP.

Wormuth et al. (2006) explored routes of exposure of the European populations to phthalates via modeling. Potential routes considered in the study included consumption of contaminated food, ingestion of contaminated soil and dust, ingestion of personal care products containing phthalates, dermal exposure to personal care products, and inhalational exposure. The authors estimated that infants and small children were exposed at the highest levels to all of the eight different phthalates studied when normalizing by body weight. The study found that dietary intake was the primary route of exposure to DEHP, DiBP, and DnBP. The main route of exposure to DEP was estimated to be dermal exposure to personal care products. This study emphasized the importance of dietary intake in total phthalate exposure as well as the wide range of routes that should be considered when assessing phthalate exposure.

The findings from our study suggest that there is an association between dietary consumption of certain food types and levels of chemical contaminants measured in the NHANES study population. Poultry consumption was significantly associated with creatinine-adjusted DEHP metabolites MEHP, MEHHP, MEOHP, and MECPP as well as high-molecular-weight phthalate metabolites. Additionally, the finding that egg consumption is significantly associated with levels of MEHP suggests that chickens themselves may be contaminated with phthalates and that food is not being contaminated just through packaging and processing. Fruit and vegetable consumption was associated with metabolites of the low-molecular-weight phthalates, such as DEP and DMP. Fruit consumption was inversely associated with metabolites of high-molecular-weight phthalates and DEHP metabolites. DEP and DMP are much more water soluble than the higher weight phthalates, which could explain their presence in fruit and vegetables (Stales et al. 1997). Meat intake was associated with the metabolite of DEP, but not with DEHP metabolites as poultry was. This finding suggests a potentially different source of contamination in poultry compared with meat. Routes of phthalate contamination for food could vary between food types, and could be from the food itself, during processing and packaging, or during storage. Because of this current uncertainty in sources of phthalate contamination in food, future studies of chemical contamination of U.S. food should involve experts from the food industry who could assist in determining at what step in the production process contamination is occurring. Studying human exposure to phthalates is complicated by the difficulty in analyzing the parent compounds because of widespread environmental contamination, including contamination in laboratories, which makes determining how much of each phthalate is present in various products challenging. Additionally, various countries have differing regulations about how much phthalate is allowed in a product, which can complicate exposure estimation. We recently described a mixture of chemicals, many with unknown or incompletely described human toxicity, found in U.S. food (Schecter et al. 2009, 2010). The potential exists for additive, synergistic, or multiplicative toxic effects for chemicals found in food or food packaging, particularly for endocrine-disrupting compounds.

Although dietary intake was significantly associated with levels of phthalate metabolites measured in urine, dietary intake, as measured in this study, cannot solely explain all of phthalate body burden. The potential routes of phthalate exposure are diverse, because of the wide range of commercial uses of phthalates. The main strengths of this study are the large sample sizes measured by NHANES, as well as the comprehensive data collected about each study participant that included both survey questions and chemical analyses of phthalate levels. The main weakness of the current approach is the cross-sectional design of the NHANES study, which makes determining causation impossible. Dietary intake in this study was estimated by a single 24-hr dietary recall that estimated the food consumed the day before urine was collected, and chemical levels were measured on only one occasion. A follow-up study could benefit from multiple phthalate metabolite measurements to provide an average level. The possibility exists that these data may not be truly representative of either dietary intake or chemical levels. However, the results from this study, combined with results from previous studies, suggest that the food supply is contaminated with phthalates, among other chemicals. A large study, possibly government sponsored, of chemical contamination of food is likely necessary to accurately assess the amount of and sources of contamination to protect the health of the public.

## Figures and Tables

**Table 1:** Sex and ethnicity of the study participants from the NHANES 2003–2004 data sets (weighted *n* = 2,350).

	Percent
Sex	
Male	48.0
Female	52.0
Ethnicity	
Mexican American	8.6
Other Hispanic	4.5
Non-Hispanic white	70.7
Non-Hispanic black	11.4
Other race	4.9

**Table 2:** Percentage of individuals underweight, normal weight, overweight, or obese from the NHANES 2003–2004 data sets (weighted *n*= 2,350).

Weight class	Percent
Underweight	9.6
Normal weight	34.1
Overweight	28.7
Obese	27.7

**Table 3:** Dietary intake of various food types for study participants in the NHANES 2003–2004 data sets (ounce equivalents consumed per day for meat, poultry, eggs, and fish; cups consumed for others).

Food type	Mean	Median	SE	Range
Total dairy	1.7	1.2	0.035	0–15.1
Total eggs	0.5	0.02	0.02	0–6.6
Total fish^a^	0.4	0	0.03	0–14.3
Total meat^b^	1.8	0.5	0.05	0–25.5
Total poultry	1.3	0	0.05	0–17.7
Total fruit	1.04	0.6	0.03	0–12.1
Legumes	0.13	0	0.01	0–3.9
Potato	0.4	0	0.01	0–9.0
Tomato	0.4	0.2	0.01	0–5.2
Total vegetables^c^	1.4	1.1	0.02	0–11.57

a Total fish low in omega-3 amino acids.

b Total meat from beef, pork, veal, lamb, and game.

c Total vegetables consumed, excluding legumes.

**Table 4:** Unadjusted levels of phthalate metabolites (ng/mL urine) measured in the NHANES 2003–2004 data set.

	Limit of detection	Median	Mean	SE	Geometric mean	Range
MECPP	0.25	35.3	90.1	6.6	34.5	0.2–2,886
MnBP	0.4	25.9	43.2	5.2	20.7	0.3–5,143
MCPP	0.16	3.3	5.6	0.3	2.9	0.1–389
MEP	0.4	207.9	675.5	69.4	194.4	3.5–46,347
MEHHP	0.32	23.1	65.2	5.1	21.8	0.2–3,141
MEHP	0.90	2.1	8.1	0.9	2.3	0.6–718
MiBP	0.26	4.9	7.1	0.6	3.7	0.2–359
MnMP	1.0	1.4	5.4	0.9	1.8	0.7–1,170
MEOHP	0.45	15.9	42.4	2.9	14.4	0.4–1,953
MBzP	0.11	17.4	29.7	1.2	13.7	0.1–1,197
Low-molecular-weight metabolites		212	680.9	70.1	201.8	4.2–46,358
High-molecular-weight metabolites		157.2	291.3	17.5	137.4	2.1–6,616
Total phthalates		458	972.2	75.3	437.9	8.9–47,585

**Table 5:** Creatinine-adjusted levels of phthalate metabolites (μg/g creatinine) measured in the NHANES 2003–2004 data set.

	Median	Mean	SE	Geometric mean	Range
MECPP	28.5	68.9	4.6	32.6	0.06–2,075
MnBP	20.7	34.0	3.5	19.6	0.09–3,596
MCPP	2.7	4.5	0.3	2.7	0.03–426
MEP	175.9	561.9	60.6	184.3	1.9–29,986
MEHHP	18.3	48.9	2.8	20.4	0.06–1,578
MEHP	1.9	6.5	0.7	2.2	0.09–294
MiBP	3.9	5.4	0.4	3.5	0.06–140
MnMP	1.5	5.0	0.8	1.7	0.2– 887
MEOHP	12.4	31.6	1.5	13.6	0.1–1,028
MBzP	13.6	22.9	0.9	12.9	0.03– 596
Low-molecular-weight metabolites	181.8	566.8	61.1	190.5	2.1–29,987
High-molecular-weight metabolites	122.3	222.6	10.6	129.7	0.7–4,901
Total phthalates	374.7	789.5	63.5	413.3	2.8–30,119

**Table 6:** Change of log-transformed creatinine-adjusted phthalate metabolite levels per additional ounce equivalent of meat, egg, poultry, or fish, or per cup of other foods, based on statistically significant bivariate and multiple regression analyses.

		Bivariate analysis		Multiple regression analysis^a^	
Dietary intake group	Phthalate metabolite	B (95% CI)	*p*-Value	B (95% CI)	*p*-Value
Dairy	MCPP	0.051 (0.033 to 0.069)	< 0.0001	0.039 (0.021 to 0.057)	0.0003
Egg	MEHP	0.13 (0.039 to 0.222)	0.0085	0.145 (0.057 to 0.232)	0.0031
Low omega-3 fish	MiBP	0.037 (0.008 to 0.066)	0.016	0.04 (0.008 to 0.073)	0.0179
Meat	MEP	0.035 (0.003 to 0.067)	0.0358	0.04 (0.006 to 0.073)	0.0224
	Low molecular weight^b^	0.034 (0.001 to 0.067)	0.0418	0.039 (0.005 to 0.072)	0.0254
Poultry	MEHHP	0.054 (0.028 to 0.08)	0.0006	0.046 (0.018 to 0.074)	0.0031
	MEHP	0.084 (0.059 to 0.11)	< 0.0001	0.08 (0.054 to 0.106)	< 0.0001
	MEOHP	0.054 (0.029 to 0.079)	0.0004	0.046 (0.02 to 0.073)	0.002
	MECPP	0.033 (0.009 to 0.057)	0.0094	0.029 (0.003 to 0.054)	0.0325
	High molecular weight^c^	0.029 (0.007 to 0.051)	0.0121	0.025 (0.003 to 0.047)	0.0294
	DEHP metabolites^d^	0.055 (0.03 to 0.081)	0.0003	0.048 (0.021 to 0.075)	0.0018
Fruit	MnMP	0.067 (0.017 to 0.116)	0.0115	0.058 (0.01 to 0.106)	0.0211
	MEHHP	−0.069 (−0.116 to −0.022)	0.0072	−0.050 (−0.09 to −0.01)	0.0173
	MEOHP	−0.066 (−0.115 to −0.017)	0.0117	−0.047 (−0.09 to −0.005)	0.0309
	MECPP	−0.050 (−0.09 to −0.011)	0.0153	−0.035 (−0.069 to −0.001)	0.0459
	MBzP	−0.091 (−0.129 to −0.052)	0.0001	−0.078 (−0.117 to −0.04)	0.0006
	High molecular weight^c^	−0.064 (−0.101 to −0.028)	0.0019	−0.052 (−0.082 to −0.023)	0.0018
	DEHP metabolites^d^	−0.066 (−0.113 to −0.018)	0.0100	−0.048 (−0.088 to −0.008)	0.0222
Potato	MEP	0.100 (0.002 to 0.198)	0.0469	0.124 (0.012 to 0.235)	0.0318
Tomato	MEP	0.148 (0.039 to 0.257)	0.0113	0.189 (0.056 to 0.321)	0.0083
	MBzP	−0.149 (−0.277 to −0.02)	0.0260	−0.147 (−0.285 to −0.009)	0.0382
	Low molecular weight^b^	0.146 (0.038 to 0.253)	0.0112	0.187 (0.057 to 0.317)	0.0079
Total vegetable	MEP	0.130 (0.063 to 0.197)	0.0009	0.142 (0.072 to 0.213)	0.0006
	Low molecular weight^b^	0.125 (0.059 to 0.191)	0.0011	0.138 (0.068 to 0.207)	0.0008
	Total phthalates	0.061 (0.012 to 0.109)	0.0181	0.073 (0.021 to 0.125)	0.0094

CI, confidence interval.

a Adjusted for BMI, age, ethnicity, and sex.

b Low-molecular-weight phthalate metabolites (sum of MEP and MnMP).

c High-molecular-weight phthalate metabo-lites (sum of MECPP, MnBP, MCPP, MEHHP, MEHP, MiBP, MeOHP, and MBzP).

d DEHP metabolites (sum of MECPP, MEHHP, MEHP, MEOHP).
